# Second harmonic generation microscopy reveals altered collagen microstructure in usual interstitial pneumonia versus healthy lung

**DOI:** 10.1186/s12931-015-0220-8

**Published:** 2015-05-27

**Authors:** Robert Matthew Kottmann, Jesse Sharp, Kristina Owens, Peter Salzman, Guang-Qian Xiao, Richard P. Phipps, Patricia J. Sime, Edward B. Brown, Seth W. Perry

**Affiliations:** Department of Medicine, University of Rochester, Rochester, NY USA; Department of Biomedical Engineering, University of Rochester, Rochester, NY USA; Department of Biostatistics and Computational Biology, University of Rochester, Rochester, NY USA; Department of Pathology and Laboratory Medicine, University of Rochester, Rochester, NY USA; Department of Environmental Medicine, University of Rochester, Rochester, NY USA

**Keywords:** Second harmonic generation, SHG, Fibrosis, Collagen, Matrix, Lung, Pulmonary, Two photon, Fluorescence, Microscopy, Usual interstitial pneumonia, Idiopathic pulmonary fibrosis, Cryptogenic organizing pneumonia

## Abstract

**Background:**

It is not understood why some pulmonary fibroses such as cryptogenic organizing pneumonia (COP) respond well to treatment, while others like usual interstitial pneumonia (UIP) do not. Increased understanding of the structure and function of the matrix in this area is critical to improving our understanding of the biology of these diseases and developing novel therapies. The objectives herein are to provide new insights into the underlying collagen- and matrix-related biological mechanisms driving COP versus UIP.

**Methods:**

Two-photon second harmonic generation (SHG) and excitation fluorescence microscopies were used to interrogate and quantify differences between intrinsic fibrillar collagen and elastin matrix signals in healthy, COP, and UIP lung.

**Results:**

Collagen microstructure was different in UIP versus healthy lung, but not in COP versus healthy, as indicated by the ratio of forward-to-backward propagating SHG signal (F_SHG_/B_SHG_). This collagen microstructure as assessed by F_SHG_/B_SHG_ was also different in areas with preserved alveolar architecture adjacent to UIP fibroblastic foci or honeycomb areas versus healthy lung. Fibrosis was evidenced by increased col1 and col3 content in COP and UIP versus healthy, with highest col1:col3 ratio in UIP. Evidence of elastin breakdown (i.e. reduced mature elastin fiber content), and increased collagen:mature elastin ratios, were seen in COP and UIP versus healthy.

**Conclusions:**

Fibrillar collagen’s subresolution structure (i.e. “microstructure”) is altered in UIP versus COP and healthy lung, which may provide novel insights into the biological reasons why unlike COP, UIP is resistant to therapies, and demonstrates the ability of SHG microscopy to potentially distinguish treatable versus intractable pulmonary fibroses.

## Background

Pulmonary fibrosis results from accumulation of fibroblasts, scar-forming myofibroblasts, and extracellular matrix proteins including collagen, often leading to irreversible loss of lung function. It can be caused by various factors including toxins, radiation exposure, autoimmune disorders, and infection. Idiopathic Pulmonary Fibrosis (IPF) is a severe form of fibrotic lung disease that can progress to respiratory failure and has a prognosis worse than lung cancer. There are currently few effective therapies. Usual interstitial pneumonia (UIP) is the histopathology underlying IPF and is characterized by heterogeneity of disease and accumulation of fibroblast foci and collagen with an emphasis on collagen type I (col1) over type III (col3) [1, 2], and abnormalities in other matrix molecules including elastin [3]. IPF is one of many diseases associated with significant collagen and other matrix protein accumulation. It is the most common of the idiopathic interstitial pneumonias, is increasing in prevalence, and it is a progressive disease that causes significant morbidity and mortality. The median duration of survival from the time of diagnosis is only 2.9 years [4, 5]. There are currently few effective FDA approved treatments for IPF (for review and overview of current and targeted therapies for IPF, please see: [6–10]), making research into IPF pathogenesis critical.

Cryptogenic organizing pneumonia (COP) is another common fibrotic lung disease. It is also characterized by accumulation of matrix components resulting in organized areas of granulation tissue in the lung. Components of this pathologic matrix accumulation in COP also include col1 and col3 (with an emphasis on col3 over col1, in contrast to UIP), fibronectin, and proteoglycans [1, 11]. In contrast to UIP, the granulation tissue found in COP accumulates in the airspaces and small airways rather than in the interstitial spaces and importantly, COP is a treatable disease with most cases responding to corticosteroids. Although the matrix components of UIP and COP have some similarities, it is unknown why the excess matrix in COP can be reabsorbed or cleared after treatment with corticosteroids while the matrix in UIP is resistant to treatment and resolution [1].

A growing body of literature supports the roles of matrix organization and structure as important effectors of fibrotic lung disease. Extracellular matrix (ECM) components have important mechanobiological properties including the abilities to activate pro-fibrotic cytokines; regulate cell trafficking; and modulate cell activation, proliferation, survival and differentiation [12, 13]. The organization and structure of the ECM, including collagen, also helps regulate availability of and interactions with a large variety of cell-matrix binding sites critical for controlling lung function. These findings further reinforce the notion that in biology, structure is a key determinant of function. Indeed, other data suggests that ECM stiffness regulates key cellular activities that may contribute to IPF [14], as well as endogenous lung function [15]. Hence, there is heightened interest in the content and structure of the matrix, and how abnormal content and structure may impact lung pathophysiology. For these reasons, we hypothesized that differences in ECM structure, and collagen microstructure in particular, underlie the different natural histories, prognoses, and responses to treatment of UIP and COP.

To explore this question, we used Second Harmonic Generation (SHG) Microscopy (SHGM) to compare the matrix of UIP and COP to that of healthy lung tissue. SHGM is a variant of two photon (2P) microscopy that can detect the fibrillar collagens (FCs) without exogenous labels. The fibril-forming collagens include collagen types 1–3, 5, 11, 24, and 27 [16], and at least several of these FCs such as types I, III, and V are key players in lung fibroses including usual interstitial pneumonia (UIP) and cryptogenic organizing pneumonia (COP) [1–3, 11]. SHGM can be used to interrogate changes in collagen’s macrostructural properties (e.g. collagen fiber density, arrangement, and organization), as well as collagen’s subresolution microstructural properties (e.g. the diameter, order versus disorder, and/or packing density of collagen fibrils within larger collagen fibers) [17–22]. These microstructural features of individual collagen fibers, as they can influence SHG directionality from that fiber (i.e. F_SHG_/B_SHG_, defined below), are herein collectively referred to as collagen “microstructure”. In this aspect, SHGM is unique in its ability to interrogate subresolution structure of FCs (e.g. col1 and col3) in intact and potentially live samples without exogenous labels, abilities which also make SHGM an attractive potential clinical and investigational diagnostic tool. Thus this technique can utilize intrinsic properties of matrix components to characterize the content and organization of the ECM in these fibrotic lung diseases.

Using SHGM, herein we describe important differences in matrix content and organization in UIP/IPF and COP compared to healthy lung tissue. Specifically, we found differences in collagen’s subresolution structural properties in UIP compared to COP and healthy lung as assessed by SHGM and the F_SHG_/B_SHG_ ratio. Importantly, even adjacent normal UIP tissue exhibited these differences in collagen microstructure compared to healthy lung, thus introducing the compelling possibilities that altered collagen microstructure might lead to or correlate with fibrosis in the relatively intractable disease UIP, but not in the more treatable COP. We also report different col1:col3 ratios in UIP versus COP and healthy lung tissue, which is important especially in the context of our F_SHG_/B_SHG_ data, because others have reported that altered col1:col3 ratios can drive (or perhaps be driven by) changes in FC microstructure such as fibril diameter [23–25], which is one aspect of collagen microstructure interrogated by the F_SHG_/B_SHG_ ratio [17–22]. Finally, we show both UIP and COP have differences in mature elastin fiber content, and elastin:collagen ratio, suggesting that both fibrotic disease have identifying physiological differences in matrix structure suggestive of lung disease, but only the less tractable disease, UIP, exhibits differences in underlying collagen microstructure. These results are important because they provide new insights into the potential biological and biostructural underpinnings of refractory versus “treatable” lung fibroses, with an emphasis on subresolution collagen microstructure, and demonstrate SHGM’s potential as a powerful new tool for aiding in the diagnosis and treatment of lung fibrosis.

## Methods

### Patient populations and source of tissue

Formalin-fixed paraffin embedded human lung tissue sections were obtained from the University of Rochester Department of Pathology using an RSRB approved protocol, by database search for resected lung tissue from mixed-sex patients with pathologically confirmed diagnoses of either UIP or COP. Additional UIP lung tissue was obtained from the NIH sponsored Lung Tissue Research Consortium. All UIP and COP biopsy specimens contained established, moderate to severe fibrosis by Pathologist diagnosis. Healthy lung tissue specimens were obtained from non-smoker subjects who had a lung biopsy for a lesion that was confirmed either benign or not primary lung cancer, from regions adjacent to the lesions that did not contain any portion of the lesion.

### Histology and immunohistochemistry

Immunohistochemistry (IHC) for col1 and col3 was performed as previously described [19] and excerpted in part herein (with modifications). Briefly, formalin fixed paraffin embedded (FFPE) lung biopsies obtained as above were sectioned at 15 um, then static-mounted on positively charged slides. For IHC, sections were deparaffinized with xylene and graded ethanols, followed by 30 minutes microwaving (65 °C) in sodium citrate solution for antigen retrieval, 2 × 5 min in sterile PBS, then blocked for one hour (10 % goat serum, 0.5 % BSA, 0.2 % Triton-X, 0.3 M glycine in PBS). Primary antibodies for Collagen I (#C2456, Sigma-Aldrich, St. Louis, MO; 1:1000) and/or Collagen III (#ab7778, Abcam, Cambridge, UK; 1:200) diluted in blocking buffer, were then applied for 2 h at room temperature in a humidified chamber, followed by 3 × 5 min PBS wash, then one hour of Alexa Fluor 594-conjugated goat anti-rabbit (for Col3) or goat anti-mouse (for Col1) IgG secondary antibodies (1:500 in 2 % goat serum, 0.25 % BSA; Invitrogen, Carlsbad, CA). Optimal antibody dilutions and incubation times were pre-determined empirically. Following staining for col1 and/or col3, lung sections were washed 3 × 5 min in PBS and mounted in ProLong Gold Antifade reagent (without DAPI; Invitrogen, Carlsbad, CA), then allowed to dry before imaging. Imaging and quantification of these tissues labeled for col1 and col3 was then performed as described in “Col1/Col3 ratio imaging” below. Hematoxylin-eosin (H&E) staining was performed by standard methods as previously described [19].

### Two photon and SHG microscopy

#### F_SHG_/B_SHG_ Imaging

Formalin fixed paraffin embedded human lung tissue sections for healthy, UIP and COP were obtained and prepared on slides as described above then imaged (unstained, unless otherwise described) for forward (F_SHG_) and backward (B_SHG_) SHG signals as previously described [19, 26, 27] and as excerpted in part herein, with modifications. We [26] and others [28] have demonstrated that reliable F_SHG_/B_SHG_ data is obtained from paraffin embedded human biopsy tissues. Double-blinded samples were imaged using a custom built multiphoton microscope, with a Mai Tai titanium:sapphire laser (Newport/Spectra Physics, Santa Clara, CA) providing two-photon (2P) excitation (100 fs pulses at 80 MHz and 810 nm) which was circularly polarized by passing the beam through a Berek compensator (Model 5540, New Focus, Irvine, CA) before the scanner. Beam scanning and image acquisition were performed with a custom-modified Fluoview FV300 confocal scanner interfaced with a BX61WI upright microscope (Olympus, Center Valley, PA), with an Olympus XLUMPLFL20xW water immersion lens (20×, 0.95 N.A.) collecting the epi-directed backscattered SHG (B_SHG_) and an Olympus 0.9 N.A. optical condenser simultaneously collecting the forward-scattered SHG (F_SHG_) using HQ405/30 m-2P emission filters (Chroma, Rockingham, VT) and HC125-02 photomultiplier tubes (PMTs) (Hamamatsu Corporation, Hamamatsu, Japan) for both F_SHG_ and B_SHG_. Excitation light (810 nm) was separated from emission signals by a short pass dichroic mirror (Chroma 670 DCSX) on the backwards (B_SHG_) side, and a 565 nm long pass dichroic mirror (565 DCSX, Chroma) on the forward (F_SHG_) side. Thus F_SHG_ and B_SHG_ were simultaneously captured in two distinct channels on every scan. The resulting two-channel (F_SHG_ and B_SHG_) images were 680 microns across. Laser power was monitored and kept constant throughout each experiment and across experimental repetitions, as were PMT voltage, gain, and offset. Because the goal was to compare relative differences in F_SHG_/B_SHG_ between the experimental conditions, and all experimental conditions to be compared were imaged during each imaging session, further calibration of the PMTs (e.g. to a reference standard) was not required.

Using these methods, we obtained z-stacks (3 um steps over the entire tissue thickness) for F_SHG_ and B_SHG_ in two channels simultaneously, from 3-6 sections, ~5 random collagen-containing regions of interest (ROIs) (images)/section, and 15–30 ROIs total per patient, for N = 5, 5, and 10 Healthy, COP, and UIP patients respectively. All data was plotted as N = number of patients per group. For each channel (F_SHG_ and B_SHG_), the image stack was maximum intensity projected (which effectively “autofocuses” each Z-stack into comparable single images), then image analysis was performed with ImageJ as previously described [19, 26, 27]. Briefly, background was defined by the average pixel counts of an equivalent laser-excited maximum intensity projected image stack taken from an area of the slide with no tissue, and subtracted from the raw F_SHG_ or B_SHG_ maximum intensity projected image stacks. These background subtracted F_SHG_ and B_SHG_ images were divided to create an F_SHG_/B_SHG_ ratio image. To calculate F_SHG_/B_SHG_, a common threshold was applied to all F_SHG_/B_SHG_ ratio images to distinguish fibrillar collagen pixels from background pixels, and subthreshold background (i.e. non collagen fiber) pixels were excluded from analysis by binary masking. This average F_SHG_/B_SHG_ value from each image was averaged across all images per patient, to yield a single representative F_SHG_/B_SHG_ value for each patient, which were then expressed as mean F_SHG_/B_SHG_ ± SEM for the Healthy, COP, and UIP patient populations.

#### Col1/Col3 ratio imaging

The same patient sets or subsets as described above for F_SHG_/B_SHG_ imaging were immunofluorescently (IF) labeled for Col1 and Col3 as described in “Histology and Immunohistochemistry” above. Following this labeling with anti-Col1 and anti-Col3 antibodies, two photon imaging was performed as described for F_SHG_/B_SHG_, except now two-photon excited fluorescence (TPEF) for immunofluorescently labeled Col1 or Col3 was captured in the backwards (epidirected) channel only, with the IF signal captured with a HQ635/30 m-2P emission filter (Chroma) and HC125-01 Hamamatsu PMT. Z-stacks from each ROI were obtained, intensity projected, and background subtracted for all sections and ROIs per patient as described for F_SHG_/B_SHG_ above. Fluorescent intensities from the resultant images were quantified with ImageJ and then expressed as mean anti-Col1 or anti-Col3 IF pixel intensity ± SEM per patient, as described above and previously [19]. Col1:Col3 ratio was quantified in the same fashion, then dividing the mean Col1/Col3 signals for each patient.

#### Collagen/elastin ratio imaging

The same patient sets or subsets were imaged and quantified for total FC content (i.e. total F_SHG_ + B_SHG_ signals) and intrinsic autofluorescence (AF) from mature lung elastin (captured at 515–555 nm), as follows. Imaging was performed exactly as for F_SHG_/B_SHG_. Immediately after each simultaneous F_SHG_ and B_SHG_ stacks was taken, the backward channel filter was replaced with a 535/40 emission filter (Chroma) and a replicate stack taken, to capture intrinsic autofluorescence (AF) from mature lung elastin in exactly the same ROIs from which collagen SHG was obtained. Elastin AF, F_SHG_, and B_SHG_ images were processed as described above. This mean Elastin AF signal per patient ± SEM was quantified and expressed both by itself and relative to the total FC signal (i.e. total SHG signal, or F_SHG_ + B_SHG_).

### Statistical analyses

All data are expressed as patient means +/- SEM. A one way ANOVA with Dunnett’s post-hoc tests correcting for multiple comparisons were used to establish statistical significance using “R” (http://www.R-project.org) and GraphPad Prism (http://www.graphpad.com) software. Results were considered significant if p < 0.05.

## Results

### Fibrillar collagen microstructure in the ECM is different in UIP, but not COP, versus healthy lung

**Fig. 1:**
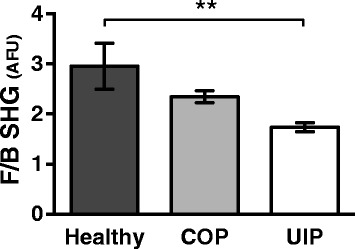
Fibrillar collagen microstructure is different in UIP, but not COP, versus healthy lung. SHG imaging was performed on healthy, COP, and UIP formalin fixed paraffin embedded human lung tissue, and the F_SHG_/B_SHG_ ratio was calculated to assess relative differences in FC microstructure. Plot represents mean F_SHG_/B_SHG_ pixel intensity ± SEM for each disease, calculated as described in Methods. Compared to healthy lung tissue, F_SHG_/B_SHG_ was significantly decreased only in UIP (**p < .0018) but not COP. Plots were generated from a total of 15–30 sections and 75–150 distinct ROIs from N = 5 patients (Healthy and COP), and 30–60 sections and 150–300 distinct ROIs from N = 10 patients (UIP). All data was plotted as N = number of patients per group. Statistics were performed by one way ANOVA with Dunnett’s post-hoc test and correction for multiple comparisons against healthy control. F/B SHG values are a ratio of mean pixel intensities in relative arbitrary fluorescent units (AFU)

**Fig. 2:**
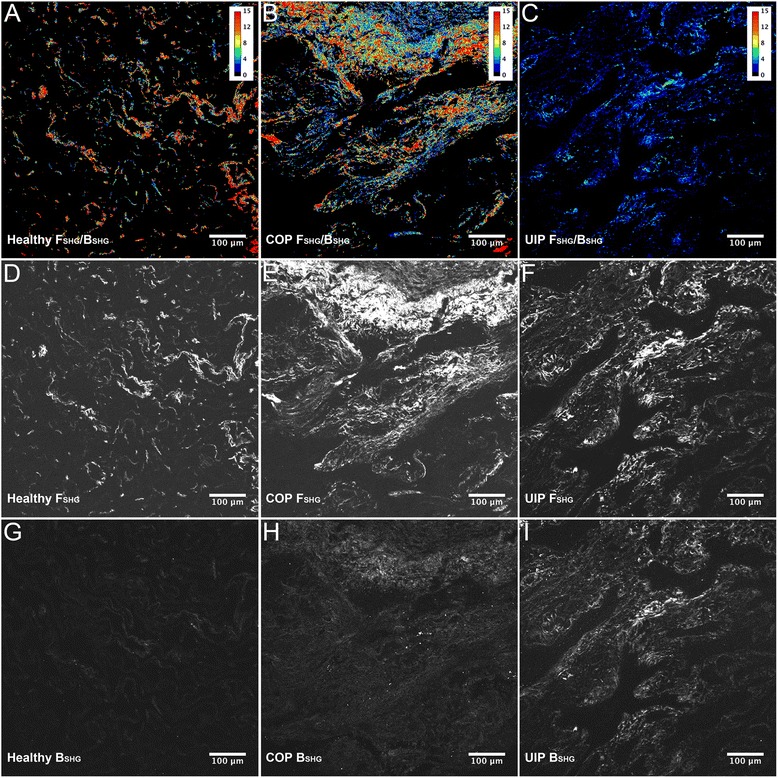
Illustration of F_SHG_, B_SHG_, and F_SHG_/B_SHG_ imaging as seen in Fig. 1. SHG imaging was performed on healthy, COP, and UIP formalin fixed paraffin embedded human lung tissue, and F_SHG_, B_SHG_, and F_SHG_/B_SHG_ ratio images obtained, as described for Fig. 1. Panels **a–c**, showing look-up-table (LUT) “heatmaps” applied to the F_SHG_/B_SHG_ ratio pixel values for representative F_SHG_/B_SHG_ images, illustrate that Healthy lung tissues have the lowest collagen content (as expected) but higher F_SHG_/B_SHG_ ratios compared to COP and UIP which evidence fibrosis and lower average F_SHG_/B_SHG_ ratios as quantified in Fig. 1. Panels **d–f** and **g–i** respectively show the corresponding F_SHG_ and B_SHG_ images for each condition, with Healthy tissue again showing the lowest collagen content and highest F_SHG_ signal intensity relative to B_SHG_ signal intensity (i.e. the highest F_SHG_/B_SHG_ ratio), whereas COP has high fibrosis and slightly higher B_SHG_ relative to F_SHG_ signals (intermediate F_SHG_/B_SHG_ ratio), and UIP also has evident fibrosis and the least differential between the F_SHG_ and B_SHG_ signal intensities (i.e. the lowest F_SHG_/B_SHG_ ratio). Levels (screen stretch) are linear and set the same for images **a–c** and **d–i**

SHG in general is sensitive to changes in collagen microstructure including regularity or ordering of collagen fibrils within larger collagen fibers; fibril compaction; and fibril diameter, tilt angle, or pitch angle [17–22, 29–34]. SHG is emitted both forwards and backwards (i.e. epi-directed) from the SHG-generating scatterers in the focal volume, and the F_SHG_/B_SHG_ ratio in particular is primarily sensitive to the spatial extent of SHG-generating scatterers along the optical axis, i.e. the effective diameter or packing arrangement/density/order versus disorder of collagen fibrils within the SHG focal volume [18–22, 26]. Therefore, to determine if a relatively intractable lung fibrosis such as UIP has a different underlying FC microstructure in the ECM versus a treatable lung fibrosis such as COP, or versus healthy lung, we used SHGM to interrogate the mean F_SHG_/B_SHG_ ratio in the ECM of UIP, COP, and healthy lung tissues. Intriguingly, we found this F_SHG_/B_SHG_ ratio was significantly decreased UIP versus healthy lung, but unchanged in COP versus healthy lung (Fig. 1). Figure 2 illustrates the results quantified in Fig. 1 with representative F_SHG_, B_SHG_, and F_SHG_/B_SHG_ images from each condition. Although corresponding clinical data such as symptoms or pulmonary function testing was not available, all patients with UIP and COP had moderate to severe pathology on the biopsy specimens. Importantly, the lack of significant variability in the F_SHG_/B_SHG_ ratio between patients in each disease group and between healthy controls suggests there is a disease specific phenotype. Additional studies will be necessary to determine whether there is a difference in the F_SHG_/B_SHG_ ratio seen in UIP on the basis of disease severity.

**Fig. 3:**
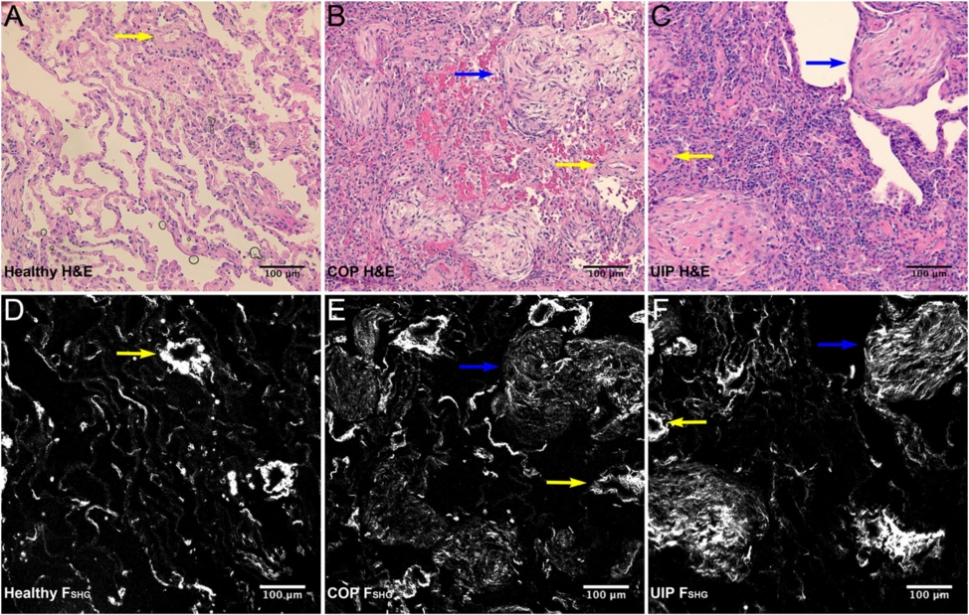
Healthy, COP, and UIP lung histopathology compared to F_SHG_. Representative hematoxylin and eosin (H&E) FOVs showing healthy **a** versus fibrotic COP **b** and UIP **c** pathology were field-matched to the F_SHG_ images **d, e, f** for the same FOVs, respectively. Note the eosin stained areas of concentrated collagen deposition (light pink color, indicated by blue arrows in **b** and **c**) that match areas of high FC F_SHG_ signal intensity (white pixel regions, indicated by blue arrows in **e** and **f**) in COP and UIP respectively. In contrast, the F_SHG_ (collagen) signal in healthy tissue **d** arises primarily from alveolar parenchyma and muscular arteries (examples of muscular arteries are indicated by yellow arrows, in all images). Thus SHGM detects and allows quantification of altered microstructure (e.g. Figs. 1, 3 and Fig. 5) from both intrinsic normal and pathologic collagen content in lung tissue. Levels (screen stretch) are linear and set the same for all images **d**–**f**

Figure 3 shows representative H&E staining (2A-C) matched to the same fields of view (FOVs) for F_SHG_ (2D-F) for healthy, COP, and UIP respectively, and illustrates that the SHG signal (white pixels, 2D-F) quantified from these lung tissues arises as expected chiefly from small airways (yellow arrows) and parenchymal alveolar space in healthy lung (2A/D), and from fibrotic collagen deposition (blue arrows) in COP (2B/E) and UIP (2C/F).

Together these results show that FC microstructure is altered in UIP but not in COP versus healthy lung.

### Lung tissue with preserved alveolar architecture adjacent to UIP fibroblastic foci or honeycomb areas has different fibrillar collagen microstructure versus healthy lung

**Fig. 4:**
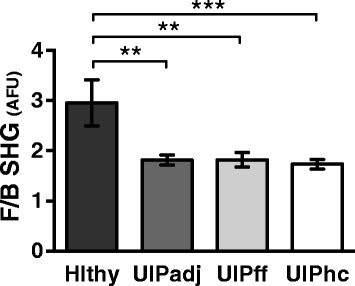
Lung tissue with preserved alveolar architecture adjacent to UIP fibroblastic foci and honeycomb areas have different fibrillar collagen microstructure versus healthy lung. SHG imaging of the F_SHG_/B_SHG_ ratio was performed on healthy (Hlthy) and UIP lung tissue in areas with preserved alveolar architecture adjacent (UIPadj) and compared to UIP fibroblastic foci (UIPff) and honeycomb regions (UIPhc). Methodology was otherwise as described in Fig. 1. The F_SHG_/B_SHG_ ratio was significantly decreased in UIPadj (**p < .0036) and UIPff (**p < .0054) and UIPhc (**p < .0007) versus healthy lung tissue – whereas UIPadj, UIPff, and UIPhc were not significantly different from each other – suggesting that even “normal appearing” lung tissue in UIP patients has altered FC microstructure. Plots were generated from a total of 15–30 sections and 75–150 distinct ROIs from N = 5 patients (Healthy and UIPff); 18–36 sections and 90–180 distinct ROIs from N = 6 patients (UIPadj); and 30–60 sections and 150–300 distinct ROIs from N = 10 patients (UIPhc). All data was plotted as N = number of patients per group. Statistics were performed by one way ANOVA with Dunnett’s post-hoc test and correction for multiple comparisons against healthy control. F/B SHG values are a ratio of mean pixel intensities in relative AFU

Next, we wondered whether lung tissue with preserved alveolar architecture adjacent to UIP fibroblastic foci or honeycomb areas also had different FC microstructure versus healthy lung as measured by F_SHG_/B_SHG_, which might suggest the possibility of underlying collagen structural deficits that could predict or predispose development of UIP. Indeed, fibroblastic foci, honeycomb areas, and surrounding normal appearing lung tissue in UIP all had similar F_SHG_/B_SHG_ ratios, which were all different versus the F_SHG_/B_SHG_ of healthy lung tissue (Fig. 4). These results provide an exciting, previously unreported “first glance” into the biologic underpinnings of UIP as relates to FC microstructure, and suggest the possibility that pre-existing alterations in FC microstructure even in “normal” lung tissue may foreshadow or precipitate development of UIP.

### Col1, Col3, and Col1/Col3 ratio differences in UIP versus COP and healthy lung

Col1 and col3 are implicated in the pathology of UIP and COP, and as fibrotic diseases, col1 and col3 levels in UIP and COP are anticipated to be higher compared to healthy lung. Moreover, previous reports have suggested that col1 is the primary collagen deposited in UIP, whereas col3 assumes this role in COP [1]. Importantly, relative col1 and col3 expression levels can interact to regulate aspects of collagen microstructure such as collagen fibril or fiber diameter [23–25]. Conversely, by altering availability of fibroblast (or other effector cell type) binding sites on collagen fibrils, changes in collagen’s subresolution fibril microstructure may regulate relative collagen expression levels. Therefore, we wished to determine how changes in F_SHG_/B_SHG_ ratio (Fig. 1), indicative of altered collagen microstructure in lung ECM, correspond with changes in col1/col3 deposition in UIP, COP, and healthy lung.

**Fig. 5:**
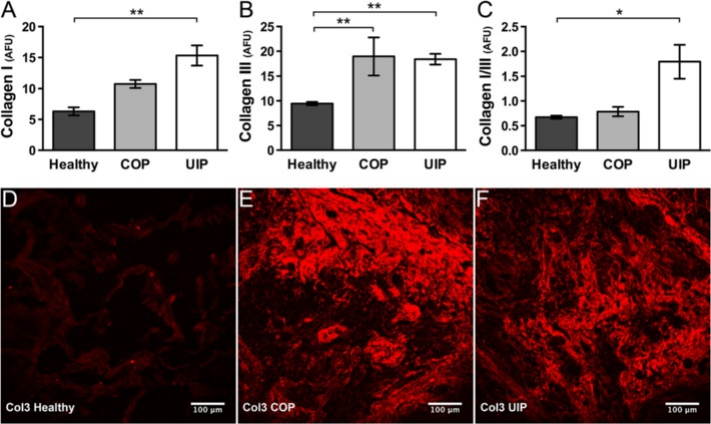
Increased Col1 and Col3 deposition, and Col1:Col3 ratio differences, in UIP or COP versus healthy lung. **a** Compared to healthy, Col1 deposition was significantly increased in UIP (**p < .0033) and trended toward an increase in COP (p = .13). **b** Col3 deposition was about equally increased in both COP (**p < .009) and UIP (**p < .004) versus healthy. Overall, this led to **c** The Col1:Col3 ratio being effectively equivalent in COP versus healthy, but significantly increased in UIP versus healthy (*p < .015). Plots were generated from ≥ 9-18 sections and ≥ 45–90 distinct ROIs from N ≥ 3 patients per condition. All data was plotted as N = number of patients per group. Statistics were performed by one way ANOVA with Dunnett’s post-hoc test and correction for multiple comparisons against healthy control. Values for Collagen I and III represent mean immunofluorescence pixel intensities in relative AFU (Panels **a**, **b**), or a ratio thereof (Panel **c**). In panels **d**–**f**, for illustrative purposes, the originally grayscale Col3 immunofluorescence is shown with “Red” LUT applied in ImageJ for Healthy, COP, and UIP respectively, with levels (screen stretch) linear and set the same for all images. Note the round Masson’s bodies characteristic of COP (in **e**), whereas **f** respresents an area of widespread late-stage fibrosis in UIP

We found higher col1 levels in both UIP and COP compared to healthy lung, with UIP showing the highest col1 levels versus COP and healthy (Fig. 5a). Both UIP and COP had similarly elevated col3 levels versus healthy lung (Fig. 5b). Overall, this resulted in relative col1:col3 ratios that were significantly elevated in UIP versus healthy controls, but not in COP versus healthy controls (Fig. 5c). Fig. 5d-f illustrate higher Col3 levels in COP (4E) and UIP (4 F) versus healthy (4D), as is shown in 4B. Together, these results demonstrate the expected evidence of fibrosis in both UIP and COP compared to healthy lung controls, and confirm previous observations of higher relative col1:col3 deposition in UIP, versus more abundant col3 over col1 deposition in COP [1].

These Col1:Col3 ratio findings are also interesting in the context of the F_SHG_/B_SHG_ differences demonstrated in Fig. 1, because altered Col1:Col3 ratios are reported to regulate collagen fibril diameter and/or structure (i.e. FC microstructure) [23–25], and accordingly UIP shows a difference in both col1:col3 ratio (Fig. 5c) and F_SHG_/B_SHG_ (i.e. FC microstructure) (Fig. 1) versus healthy lung, whereas COP shows neither a difference in col1:col3 ratio nor F_SHG_/B_SHG_ versus healthy lung. Overall, these results suggest a possible relationship between FC microstructure differences and altered col1:col3 ratios in intractable UIP fibrosis, but not in the more treatment responsive COP fibrosis.

### Elastin and elastin:collagen ratios differ in UIP and COP versus healthy lung

**Fig. 6:**
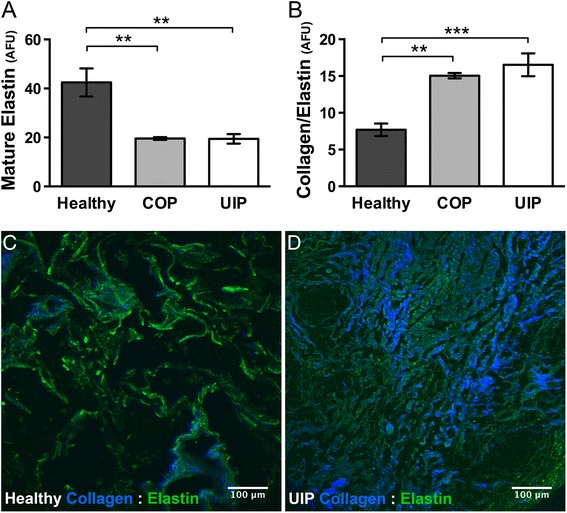
Elastin content and Collagen:Elastin ratio differ in UIP and COP versus healthy lung. **a** Mature elastin fiber content was similarly decreased in both COP (*p < .007) and UIP (*p < .004) versus healthy lung tissue, and **b** The total fibrillar collagen/mature elastin ratio was similarly increased in both COP (**p < .004) and UIP (**p < .0008) compared to healthy. Plots represent mean pixel intensity ± SEM for these elastin autofluorescence (Panel **a**) and (F_SHG_ + B_SHG_/ Elastin autofluorescence) (Panel **b**) signals captured as described in Methods, in relative AFU. Plots were generated from a total of 12–24 sections and 60–120 distinct ROIs from N = 4 patients (Healthy and UIP), and 9–18 sections and 45–90 distinct ROIs from N = 3 patients (COP). All data was plotted as N = number of patients per group. Statistics were performed by one way ANOVA with Dunnett’s post-hoc test and correction for multiple comparisons against healthy control. Representative merged images **c** and **d** illustrate this lower total FC SHG (blue):mature elastin (green) ratio as seen in **c** healthy compared to **d** UIP. (Compare total amount and intensity of the blue summed F_SHG_ + B_SHG_ collagen SHG signal, relative to the green mature elastin signal, in the healthy **c** versus UIP **d** panels respectively). Panel **d** respresents an area of widespread late-stage fibrosis in UIP. For illustrative purposes, the originally grayscale SHG and elastin fluorescence signals are shown with “Blue” and “Green” LUTs applied in ImageJ respectively, with levels (screen stretch) linear and set the same for all channels in all images

In parallel with SHGM imaging, intrinsic tissue autofluorescence representing principally mature lung elastin can be captured simultaneously with SHG [35], to provide additional insights into how ECM structure and organization may differ in UIP versus COP. Elastin is another lung ECM component that interacts closely with collagen to regulate lung function [36–39] and is frequently dysregulated in fibrotic lung diseases [40, 41]. Elastin’s intrinsic autofluorescence captured by two-photon excitation fluorescence (TPEF) microscopy arises from the pyridoxine-based pyridolamine cross-links [35, 42] found only in mature elastin fibers [43], thus making TPEF of elastin a useful indicator for the mature elastin fiber content of lung tissue. Therefore, we captured this signal for the same healthy, UIP, and COP specimens, then quantified and expressed it both by itself and relative to the total FC signal (i.e. total SHG signal, or F_SHG_ + B_SHG_), to see whether there were other underlying differences in ECM structure or organization that we could identify and quantify by SHGM and two-photon excitation fluorescence (TPEF) microscopy. Using this methodology, total mature elastin signal was similarly decreased in both UIP and COP compared to healthy lung tissue (Fig. 6a), and FC:mature elastin ratios (Fig. 6b) were similarly increased in UIP and COP compared to healthy. However, in neither of these parameters was UIP different from COP. Panels 5C-D illustrate the lower FC:mature elastin ratio seen in healthy versus UIP respectively.

These data demonstrate that compared to healthy lung, both fibrotic lung diseases (UIP and COP) are characterized by significant gross physiologic disruptions in ECM structure and organization that can be quantified with non-invasive and non-tissue destructive combined SHG and TPEF microscopy. Yet only the more intractable UIP fibrosis shows evidence of disrupted FC microstructure as interrogated by F_SHG_/B_SHG_, thus highlighting the compelling possibility that together these techniques may help make clinical distinctions between intractable and treatable lung fibroses.

## Discussion

Pulmonary fibrosis is characterized by accumulation of ECM proteins in lung tissue. The mechanisms leading to pathologic (or non pathologic) accumulation and organization of matrix proteins remain poorly understood. Although we have some insight into the composition, structure and/or organization of the matrix, many properties of the matrix remain uninvestigated. Numerous matrix proteins likely contribute to organ dysfunction in pulmonary fibrosis, however, we are only beginning to understand how homeostasis and organization of these proteins impact cellular function.

Collagen, produced and organized mainly by fibroblasts and scar-forming myofibroblasts, is one of the most abundantly studied matrix proteins. At least twenty-eight different collagen subtypes have been described to date. All collagen species contain three alpha peptide sequences forming a triple helix. Collagen type is determined by the type(s) of alpha peptides and post translational modifications, hydroxylation, and/or glycosylation. Further modification of collagen structure occurs after release into the extracellular space. Here, crosslinking and joining of the helices occur to form collagen fibrils and larger collagen fibers, and fibrosis (aberrant excess deposition of collagen) may occur.

Due to their ability to provide information on the intrinsic content and structure of collagen and other endogenous ECM proteins without exogenous labels or tissue destroying procedures (see description of SHGM and TPEF in Background and Results, respectively), there has been increasing interest in using SHGM and TPEF to provide insights into the matrix structure of healthy and diseased lung, both for fibrotic as well as non-fibrotic lung diseases [28, 36, 44–48] (and for lung cancers, not discussed herein). One group reported that combined TPEF and SHG identified “characteristic features of fibroblastic foci in human Idiopathic Pulmonary Fibrosis samples” [47], whereas another report used F_SHG_ and B_SHG_ signals to differentiate Col1 from Col3 in lung tissue from patients with chronic obstructive pulmonary disease (COPD) [48]. Also in COPD, Tjin et al. found the F_SHG_/B_SHG_ ratio was different in COPD versus non-diseased lung tissue [28]. However, reports demonstrating a diagnostic capability of quantifiable SHGM and TPEF parameters to discriminate between different clinical lung fibroses, or perhaps more importantly to provide insights into the underlying etiology or structure-function origins of lung disease, are still scarce.

In this report, we extend these works by using SHGM and TPEF imaging to identify key differences in the ECM of UIP compared to COP and healthy control lung tissue. UIP and COP were chosen because they are both characterized by increases in matrix proteins, particularly FCs, yet they have contrasting natural histories, responses to corticosteroids, and prognoses. The reasons why UIP is progressive and difficult to treat are not clear. One possible explanation is that there may be a fundamental difference in collagen’s content, structure, and/or organization in the UIP ECM that renders collagen more structurally more resistant to degradation in UIP versus COP. We tested this hypothesis using SHGM, a microscopy approach that is sensitive to the intrinsic FC organization and microstructure within the matrix, to confirm whether FC in UIP has different microstructural properties versus COP or healthy lung.

Using this approach, we have demonstrated that FC microstructure in the ECM of UIP is significantly different from FC microstructure in either COP or healthy control lung tissue, as evidenced by the F_SHG_/B_SHG_ ratio. Changes in this F_SHG_/B_SHG_ ratio suggest that there is a significant difference in the density, structure, and/or organization of FC in UIP compared to COP and healthy lung tissue, particularly with regard to the effective diameter or packing arrangement/density of collagen fibrils in the ECM [18–22, 26]. These results are compelling because while previous studies (discussed above) have elegantly demonstrated the utility of SHGM for investigating lung fibroses, or have shown expression changes in several collagen subtypes in fibrotic lung diseases, to our knowledge this is the first report of abnormalities in ECM and FC microstructure in UIP as being quantifiable and differentiable from other lung fibroses (and from healthy) by SHGM, specifically F_SHG_/B_SHG_. Still more compelling is the fact that only the intractable fibrosis (UIP) demonstrated significant differences in FC microstructure versus healthy lung, whereas the treatable fibrosis (COP) did not, thus providing compelling and to our knowledge seminal evidence that alterations in collagen’s fundamental underlying structure may contribute to whether or not pulmonary fibroses are treatment responsive. These results provide previously unavailable insights into the biological underpinnings of treatment-resistant pulmonary fibrosis, and also highlight the potential of SHGM as a novel clinical diagnostic and investigational tool for distinguishing between intractable and treatable lung fibroses.

We also found that lung tissue with preserved alveolar architecture adjacent to UIP fibroblastic lesions and honeycomb areas all have different FC microstructure (i.e. F_SHG_/B_SHG_) versus healthy lung. Moreover, there was no difference in F_SHG_/B_SHG_ between UIP fibroblastic foci, honeycomb areas, and adjacent areas with preserved alveolar architecture (Fig. 4). Together these results suggest the possibility that pre-existing alterations in FC structure even in “normal” lung tissue may foreshadow or precipitate (or at minimum, associate with) development of UIP. In other words, underlying collagen structural deficits – perhaps present in pre-UIP tissue well before the onset of UIP – might be a biomarker that predicts or predisposes development of future UIP.

As expected, both col1 and col3 were elevated in UIP and COP versus healthy lung, with col1 deposition being predominant to col3 in UIP, and vice-versa in COP, as has been previously reported [1]. These results are significant in the context of our other results reported herein because it is known that changes in FC ratios, particularly col1:col3 ratios, plays a significant role in regulating collagen fibril diameter [23–25] (one component of collagen microstructure). Similarly, by regulating the availability of fibroblast (or other effector cell type) binding sites on collagen fibrils, changes in collagen’s subresolution fibril microstructure could in turn control relative levels of FC expression. In other words, different col1:col3 ratios may in turn drive or be driven by altered collagen microstructure in UIP. Together with the earlier data, these results demonstrate that the ECM of UIP not only contains more collagen (particularly more col1) than the ECM of COP and/or healthy lung tissue, but also that there are significant differences in the subresolution microstructure of these collagen fibrils (i.e. fibril diameter, density, and/or organization, as interrogated by F_SHG_/B_SHG_) in UIP versus COP and healthy, independent of the absolute amount of collagen deposition in each disease.

Finally, we demonstrated that mature elastin content in both UIP and COP is reduced compared to healthy controls (Fig. 6). Elastin’s intrinsic autofluorescence originates from pyridoxine-based pyridolamine cross-links [35, 42] found primarily in mature elastin fibers [43], therefore TPEF of endogenous lung elastin preferentially identifies mature elastin fibers in lung tissue. These results are consistent with the concept that breakdown of mature elastin fibers in the lung, and their “replacement” with often excess deposition of immature elastin fibers and elastin precursors, is believed to contribute to reduced lung function in a variety of pulmonary diseases [49]. In other words, increased elastosis (i.e. breakdown of mature elastin fibers), as has been demonstrated for both UIP and COP [50], most likely leads to a compensatory increase in elastin production in an (ultimately unsuccessful) effort to restore the mature elastin fibers which have been lost.

Hence our results here together with these previous studies all support the concept of increased elastin turnover (i.e. synthesis and “deposition” of “immature” elastin components) consequent to breakdown and loss of mature elastin fibers in UIP and COP, with resultant deficits in pulmonary function. Indeed Enomoto et al. show a significant increase in very fragmented appearing elastin (i.e. likely to be fragmented mature elastin fibers and/or deposition of immature elastin precursors; see Fig. 1 in [51]) associated with a decline in lung function in IPF [51]. Others have also reported apparently increased elastin production, for example increased elastin gene expression and protein expression [52], as well as increased enzymatic breakdown of mature elastin in COPD and IPF [50], in these and other [53] pulmonary fibroses. Finally Eurlings et al. recently reported increased collagen and decreased elastin in aveolar and small airway walls of chronic obstructive pulmonary disease (COPD) [54], another disease with fibrotic pathology, similar to what we demonstrate herein for COP and UIP (Fig. 3 and Fig. 5).

Taken together with our findings on different FC microstructure in UIP but not COP versus healthy lung, these observations on elastin content are especially compelling because they demonstrate that compared to healthy lung, both fibroses (UIP and COP) have significant identifying physiologic disruptions in ECM structure and organization that are quantifiable with non-invasive and non-tissue destructive combined SHG and TPEF microscopy. Yet only the more intractable UIP fibrosis has disrupted FC microstructure identifiable by F_SHG_/B_SHG_, and thus together these techniques may represent novel clinical diagnostic tools for distinguishing between intractable and treatable lung fibroses. The continual pre-clinical advancement of SHG and TPEF endoscopic technology makes the prospect of such a diagnostic tool for distinguishing between intractable and treatable lung fibroses all the more compelling [17, 55].

While both UIP and COP are fibrotic diseases, UIP is a fatal disease affecting the alveolar walls and subpleural areas, while COP is a treatable disease affecting the alveolar spaces and bronchiolar lumen. Thus while both have fibrosis, their topography and natural history are distinct, and therefore any inter-disease comparative study of this nature cannot completely exclude the possibility that some measured differences might be attributable at least in part in part to temporal or anatomical/topographical differences in the lung tissues examined between the patient groups. Moreover, incorporating additional corresponding clinical data such as symptoms or pulmonary function testing (unavailable for these current patient sets) will allow us to strengthen our findings and interpretations in future studies. Nonetheless the possibility that non-invasive and non-tissue destructive combined SHG and TPEF microscopy, utilized either ex vivo or perhaps ultimately in vivo, may be able to distinguish and/or predict onset or outcome of tractable versus fatal lung fibroses remains compelling.

In summary, using SHG and TPEF microscopy, herein we identify several previously unreported key differences between UIP, COP and healthy lung tissue. The collagen microstructure differences we observed in UIP ECM provide novel insights as to why this pathology may be resistant to many therapies. For example, an ECM and/or collagen fibrils that are more densely packed, more ordered or disordered, and/or more cross-linked may be more resistant to homeostatic turnover and exhibit differences in matrix stiffness that are key to modifying cellular activity of resident cells and activation of pro-fibrogenic cytokines such as transforming growth factor beta (TGF- β. Identifying all the microstructural changes present in UIP and/or the mechanisms that regulate them will be a critical part of our future research. These ongoing studies will seek to determine more specifically exactly what features of collagen’s microstructure (e.g. fibril diameter, fibril density, and/or hetero- or homo-typic fibril composition or organization) are different in UIP versus COP and healthy lung, and identify molecular targets that may effect these changes in collagen’s underlying microstructure. Although further studies are required to ascertain whether or not the altered FC microstructure as we demonstrate here is an underlying cause of (rather than just associated with) differences in natural history, treatment responsiveness, and/or prognosis between UIP and COP, at minimum these results introduce the intriguing possibility of using SHG microscopy as a novel clinical biomarker that may help predict treatment responsiveness of idiopathic fibrotic lung disease.

## Conclusions

To date it is unknown why some lung fibroses respond well to therapies, yet others remain relatively intractable. Herein, we report differences in collagen’s subresolution structure or organization (i.e. “microstructure”) and airway matrix structure in usual interstitial pneumonia versus cryptogenic organizing pneumonia and healthy lung, as identified by quantifiable nonlinear SHG and TPEF microscopy parameters. These findings may offer key insights into the biologic underpinnings of refractory versus treatable pulmonary fibroses, and highlight the potential of second harmonic generation microscopy as a novel diagnostic tool for distinguishing between these clinical scenarios.
